# Mechanical Behavior and Response Mechanism of Short Fiber-Reinforced Polymer Structures Under Low-Speed Impact

**DOI:** 10.3390/ma18153686

**Published:** 2025-08-06

**Authors:** Xinke Xiao, Penglei Wang, Anxiao Guo, Linzhuang Han, Yunhao Yang, Yalin He, Xuanming Cai

**Affiliations:** 1 Henan International Joint Laboratory of Dynamics of Impact and Disaster of Engineering Structures, Nanyang Institute of Technology, Nanyang 473004, China; xiaoxk@nyist.edu.cn; 2 School of Aerospace Engineering, North University of China, Taiyuan 030051, Chinaguoanxiao2025@163.com (A.G.); h13082018371@163.com (L.H.); yhyang0908@163.com (Y.Y.);

**Keywords:** short fiber-reinforced polymer, 3D printing, damage mode, energy dissipation

## Abstract

Short fiber-reinforced polymer (SFRP) has been extensively applied in structural engineering due to its exceptional specific strength and superior mechanical properties. Its mechanical behavior under medium strain rate conditions has become a key focus of ongoing research. A comprehensive understanding of the response characteristics and underlying mechanisms under such conditions is of critical importance for both theoretical development and practical engineering applications. This study proposes an innovative three-dimensional (3D) multiscale constitutive model that comprehensively integrates mesoscopic fiber–matrix interface effects and pore characteristics. To systematically investigate the dynamic response and damage evolution of SFRP under medium strain rate conditions, 3D-printed SFRP porous structures with volume fractions of 25%, 35%, and 45% are designed and subjected to drop hammer impact experiments combined with multiscale numerical simulations. The experimental and simulation results demonstrate that, for specimens with a 25% volume fraction, the strain rate strengthening effect is the primary contributor to the increase in peak stress. In contrast, for specimens with a 45% volume fraction, the interaction between damage evolution and strain rate strengthening leads to a more complex stress–strain response. The specific energy absorption (SEA) of 25% volume fraction specimens increases markedly with increasing strain rate. However, for specimens with 35% and 45% volume fractions, the competition between these two mechanisms results in non-monotonic variations in energy absorption efficiency (EAE). The dominant failure mode under impact loading is shear-dominated compression, with damage evolution becoming increasingly complex as the fiber volume fraction increases. Furthermore, the damage characteristics transition from fiber pullout and matrix folding at lower volume fractions to the coexistence of brittle and ductile behaviors at higher volume fractions. The numerical simulations exhibit strong agreement with the experimental data. Multi-directional cross-sectional analysis further indicates that the initiation and propagation of shear bands are the principal drivers of structural instability. This study offers a robust theoretical foundation for the impact-resistant design and dynamic performance optimization of 3D-printed short fiber-reinforced polymer (SFRP) porous structures.

## 1. Introduction

Short fiber-reinforced polymer (SFRP) composites have been widely adopted in the aerospace, biomedical, and transportation industries due to their high specific strength, high specific modulus, and exceptional structural design flexibility [1,2,3,4]. However, conventional composite manufacturing processes encounter significant challenges, such as high mold fabrication costs and limited formability for complex geometries. In contrast, 3D printing technology provides an innovative approach for customized SFRP fabrication by leveraging its mold-free and freeform capabilities [5,6,7]. It is worth noting that the mechanical behavior of 3D-printed SFRP is governed by multi-scale factors including the fiber volume fraction, spatial orientation distribution, and porosity [8,9,10], yet existing constitutive models are insufficient to accurately predict their nonlinear dynamic response. This limitation becomes particularly critical under medium strain rate service conditions, where the mechanical response characteristics and underlying mechanisms remain insufficiently understood. Therefore, a systematic investigation into the mechanical behavior and intrinsic mechanisms of 3D-printed SFRP under medium strain-rate loading is essential.

Notable advancements have been achieved in predicting the mechanical behavior of 3D-printed short fiber-reinforced polymer (SFRP) composites; yet persisting challenges continue to hinder comprehensive modeling capabilities. For instance, it has been demonstrated that carbon fiber/glass fiber-reinforced polyether ether ketone (CF/GF-PEEK) composites fabricated via extrusion molding exhibit a superior surface morphology, reduced micro-porosity, and significantly enhanced mechanical performance at low short carbon fiber volume fractions. In contrast, increasing the fiber content to higher levels results in a non-uniform microstructure marked by elevated porosity [11]. Further studies reveal that an elevated fiber content amplifies interfacial bonding heterogeneity, whereas random fiber agglomeration contributes to a spatially non-uniform distribution within the matrix. Similarly, an investigation of short glass fiber-reinforced polymer composites prepared by electric field-induced orientation technology has shown that the distribution of fiber orientation angles has a significant impact on the fracture behavior of the material [12]. When the fibers are oriented perpendicularly to the crack propagation plane, the composites exhibit higher fracture toughness; the fracture toughness of samples with randomly oriented fibers is significantly reduced; and the fracture toughness of samples with fibers oriented parallel to the crack propagation plane is the lowest. Research indicates that in the additive manufacturing of variable stiffness composites, process optimization guided by numerical simulations and based on bio-inspired wood node structures enables precise placement of curved fibers [13]. By programming the nozzle trajectory and integrating an adaptive feeding system, a non-uniform fiber distribution can be achieved without interference, gaps, or overlaps. Moreover, incorporating topological optimization allows the reinforcement architecture to transition from conventional unidirectional layouts to curved fiber configurations tailored to non-uniform stress fields, thereby substantially enhancing the utilization efficiency of fiber materials. Additionally, research shows that the in-plane raster angle of polylactic acid (PLA) materials prepared by the fused deposition modeling (FDM) process has a significant impact on tensile strength and fracture toughness [14]. PLA materials exhibit obvious anisotropy in tensile and fracture properties, among which the grating angle is a key parameter for regulating mechanical responses. When the grating direction is 45°/−45°, the material demonstrates the best fracture elongation and anti-fracture performance. Despite these advancements, predictive models for dynamic mechanical behavior still face significant limitations. A study [15] found that the mechanical properties of PLA materials manufactured by FDM 3D printing are affected by the relationship between layer thickness, raster angle, and build direction. The results showed that the optimal impact strength is achieved at a 0° raster angle and a build direction parallel to the loading direction. Another systematic study on PLA–graphene composites found that increasing the layer thickness can significantly improve tensile strength, flexural strength, and impact toughness [16]. Additionally, scholars [17] prepared tensile samples and lattice structures of PLA using FDM. Through tensile and compression experiments, they confirmed that printing temperature and printing speed affect the mechanical properties of 3D-printed PLA lattice structures: with the increase in printing temperature, tensile strength and the elastic modulus first increase and then decrease; and the material’s properties are improved at higher printing speeds. Most studies depend on experimental characterization or two-dimensional planar models, which are unable to capture the influence of three-dimensional fiber spatial distributions. As additive manufacturing progresses toward precise control of the three-dimensional fiber orientation [18], the development of high-resolution three-dimensional mechanical models becomes crucial to overcome the inherent constraints of conventional two-dimensional approaches.

In this study, a three-dimensional multiscale constitutive model is developed that incorporates fine-scale fiber–matrix interfacial effects and pore defect characteristics. Gradient volume fraction porous SFRP structures are designed and fabricated, and multiscale numerical simulations are integrated with systematic experimental validations to reveal the dynamic response mechanisms and energy absorption behaviors under impact loading conditions. Furthermore, the critical conditions and path-dependent features of damage evolution within porous structures are elucidated.

## 2. Experimental Design and Theoretical Foundation

### 2.1. Fundamentals and Principles of the Drop-Weight Test

The drop-weight impact test is a widely recognized experimental method employed to investigate the capacity of materials to sustain large deformations under impact loading. In this study, a drop-weight impact testing system was utilized to perform loading experiments on SFRP porous structures under medium strain rate conditions. The operational principle of the system [19] is as follows: a heavy hammer is released from a predetermined height, converting gravitational potential energy into kinetic energy, which subsequently applies an impact load to the specimen, inducing both impact force and corresponding deformation.

**Figure 1:**
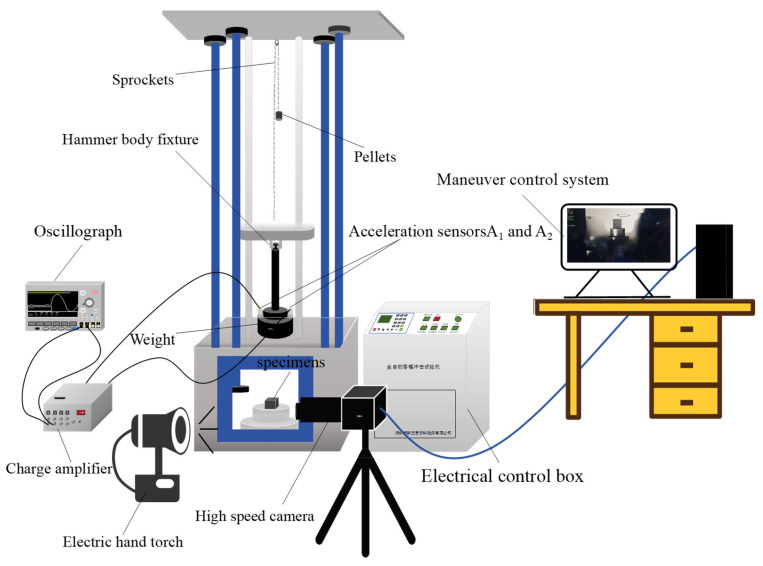
Drop-hammer-type impact testing machine.

The schematic diagram of the drop-weight impact test system is presented in Figure 1. Tests were performed at drop heights of 70 cm, 100 cm, and 120 cm. An accelerometer was attached to the upper surface of the weight to measure acceleration. The acquired signals were then transmitted to a data acquisition system for analysis and processing. The accelerometer employed was an LK111-03 (Keyence Corporation, Osaka, Japan) charge-type model (sensitivity: 12.12 pC/(m/s^2^)). The charge signal was amplified and converted into a voltage signal using an LK1432B(Shandong Yuanchuang Instruments Co., Ltd., Jining, China) charge amplifier through its feedback capacitor. A Tektronix MDO3024 (Tektronix (China) Co., Ltd., Shenzhen, China) mixed-domain oscilloscope captured the voltage signal and displayed its waveform over time. This allowed for the extraction of key acceleration signal characteristics, such as amplitude and frequency [20,21]. To minimize random errors, multiple replicate tests were conducted for each volume fraction of the SFRP porous structure under every prescribed strain rate condition. At least three valid replicates were obtained for each experimental setup to ensure statistical reliability.

### 2.2. Schwarz Primitive Porous Structure Design

**Figure 2:**
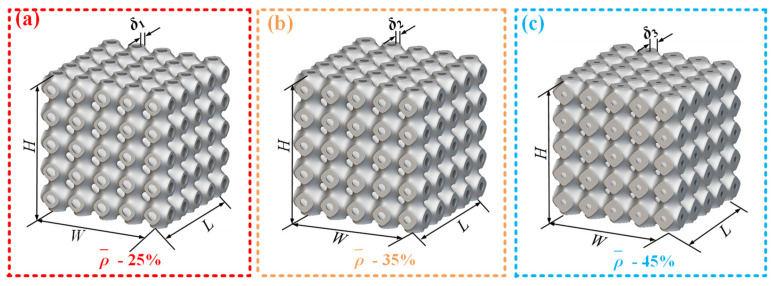
Three volume fractions of Schwarz primitive porous structure. (**a**) Schwarz primitive porous structure with 25% volume fraction(single-cell wall thickness 0.438 mm); (**b**) Schwarz primitive porous structure with 35% volume fraction(single-cell wall thickness 0.613 mm); (**c**) Schwarz primitive porous structure with 45% volume fraction(single-cell wall thickness 0.787 mm).

To evaluate the application of the SFRP fine–macro mechanical method analysis framework in characterizing composite structural properties, a three-periodic minimal surface (TPMS) porous structure was selected as the study object. The TPMS structure was designed using the Lattice_Karak (*v* 1.0) (Hyderabad, Telangana, India) software developed by Raju [22]. During the design process, the built-in Schwarz Primitive trigonometric function of the software was employed. Key parameters were established, including the wall thickness (δ) of individual unit cells, the unit cell size (6 mm × 6 mm × 6 mm), and the number of cells along each spatial direction (5 cells in X, Y, and Z) [23]. Figure 2 illustrates three Schwarz primitive porous structures with varying volume fractions, each with dimensions of 6 mm × 6 mm × 6 mm. These structures exhibit volume fractions of 25%, 35%, and 45%, corresponding to single-cell wall thicknesses (δ) of 0.438 mm, 0.613 mm, and 0.787 mm, respectively. The overall dimensions of the assembled TPMS structures are 30 mm × 30 mm × 30 mm.

The specimens were fabricated using the fused deposition molding (FDM) technique with a Bambu Lab X1 printer (Shenzhen Tuo Zhu Technology Co., Ltd., Shenzhen, China) and CF-PLA composite (Banbu lab Inc, shenzhen, China) wire as the primary material. The process parameters of Bambu Lab X1 are detailed in Table 1. Post-processing was subsequently conducted to relieve residual internal stresses. This was accomplished by drying the specimens in a SUPO (Shaoxing Supo Instrument Co., Ltd., Shaoxing, China) electrothermal constant-temperature blast drying oven. After printing, the specimens were manually detached from the printing substrate and carefully placed into the oven for thermal treatment. Once the drying process was completed, the specimens were labeled and prepared for subsequent experimental analyses.

The 3D model overcomes the spatial limitations inherent in 2D models, enabling full characterization of multi-axial stress states. Traditional 2D methods (e.g., plane strain or plane stress models) fundamentally assume that “fibers and loads are confined to a 2D plane”. Consequently, they can only capture in-plane stress components (*σ*ₓ, *σ*_y_, and *τ*_xy_), completely neglecting the out-of-plane normal stress (*σ*_z_) and out-of-plane shear stresses (*τ*_xz_ and *τ*_yz_) that inevitably exist in 3D space. This simplification introduces significant limitations in analyzing the dynamic impact response of porous structures. Regarding the 3D fiber orientation effects, in 3D-printed SFRP, fibers are randomly distributed in space (micro-CT observations reveal that approximately 30% of fibers exhibit inclinations of 15–30° relative to the z-axis). Two-dimensional models forcibly project these fibers onto the x-y plane, underestimating their load-bearing contribution. In contrast, the 3D model directly incorporates the 3D coordinates and orientation angles of fibers. It calculates anisotropic stiffness using micromechanical formulas (e.g., the modified Tsai–Hill criterion), accurately reflecting the reinforcement effect of z-direction fibers on longitudinal load-bearing. Otherwise, under a medium strain rate impact, shear bands in porous structures do not propagate solely within the x-y plane; instead, they nucleate along 3D oblique planes. Two-dimensional models can only predict in-plane shear failure, failing to capture these spatially inclined shear bands, which leads to prediction errors exceeding 25% in failure initiation locations. The 3D model, by tracking 3D stress gradients, accurately simulates the helical propagation path of shear bands from the surface to the interior, showing excellent agreement with experimentally observed damage morphologies.

The 3D model precisely characterizes the three-dimensional nature of the microstructure, avoiding the physical mechanism distortion caused by 2D simplification. The pores in Schwarz primitive porous structures are fully interconnected in 3D (Figure 2). Under impact loading, energy absorption occurs through simultaneous compression along the x, y, and z directions. Two-dimensional models simplify these to planar pores, only simulating compression in the x-y directions while neglecting the energy absorption contribution from z-direction pore collapse. This results in an underprediction of specific energy absorption compared to experiments. In contrast, the model calculates real-time 3D pore volume changes, limiting the prediction error for specific energy absorption to within 5%.

### 2.3. Macroscopic Failure Criteria and Damage Evolution Models

The tensile and compressive strengths of short fiber-reinforced composites are generally unequal along the principal material direction. This discrepancy becomes particularly evident in the transverse direction, where significant differences exist between tensile and compressive strengths [24,25,26]. Based on these findings, Tsai and Wu proposed the Tsai–Wu tensor failure criterion in 1971, which is also known as the stress space failure criterion [27]. The macroscopic equivalent structure of short fiber-reinforced composites can be modeled as a homogeneous anisotropic material. This modeling approach justifies the applicability of the Tsai–Wu tensor failure criterion. The mathematical formulation of the Tsai–Wu tensor failure criterion is expressed as follows:

(1) $$F_{11}\sigma_{1}^{2}+F_{22}\sigma_{2}^{2}+F_{66}\sigma_{6}^{2}+2F_{12}\sigma_{1}\sigma_{2}+F_{1}\sigma_{1}+F_{2}\sigma_{2}=1$$

(2) $$\begin{array}{l} F_{11}=\frac{1}{X_{t}X_{c}},F_{22}=\frac{1}{Y_{t}Y_{c}}\\ F_{1}=\frac{1}{X_{t}}-\frac{1}{X_{c}},F_{2}=\frac{1}{Y_{t}}-\frac{1}{Y_{c}}\\ F_{66}=\frac{1}{S^{2}},-\frac{1}{2}\sqrt{F_{11}F_{22}}\leq F_{12}\leq 0 \end{array}$$

where *X*_t_ is the longitudinal tensile strength, *X*_c_ is the longitudinal compressive strength, *Y*_t_ is the transverse tensile strength, *Y*_c_ is the transverse compressive strength, and *S* is the shear strength.

The equivalent constitutive relationship for the macroscopic model of SFRP is expressed as follows [28]:

(3) $$\left\{\begin{array}{c} \sigma_{1}\\ \sigma_{2}\\ \sigma_{3}\\ \tau_{23}\\ \tau_{31}\\ \tau_{12} \end{array}\right\}=\left[\begin{array}{cccccc} C_{11} & C_{12} & C_{13} & 0 & 0 & 0\\ C_{12} & C_{22} & C_{23} & 0 & 0 & 0\\ C_{13} & C_{23} & C_{33} & 0 & 0 & 0\\ 0 & 0 & 0 & C_{44} & 0 & 0\\ 0 & 0 & 0 & 0 & C_{55} & 0\\ 0 & 0 & 0 & 0 & 0 & C_{66} \end{array}\right]\left\{\begin{array}{c} \varepsilon_{1}\\ \varepsilon_{2}\\ \varepsilon_{3}\\ \gamma_{23}\\ \gamma_{31}\\ \gamma_{12} \end{array}\right\}$$

Its stiffness matrix has nine independent stiffness coefficients: *C*_11_, *C*_12_, *C*_13_, *C*_23_, *C*_22_, *C*_33_, *C*_44_, *C*_55_, and *C*_66_. The relationship between the stiffness coefficients and the engineering elastic constants of the equivalent materials of the macroscopic model can be expressed as follows:

(4) $$\left\{\begin{array}{c} \begin{array}{cc} C_{11}=\frac{1-v_{23}v_{32}}{E_{2}E_{3}\Gamma }, & C_{12}=\frac{v_{21}+v_{23}v_{31}}{E_{1}E_{3}\Gamma } \end{array}\\ \begin{array}{cc} \;C_{13}=\frac{v_{31}+v_{21}v_{32}}{E_{1}E_{2}\Gamma }, & C_{23}=\frac{v_{32}+v_{12}v_{31}}{E_{1}E_{2}\Gamma } \end{array}\\ \begin{array}{cc} C_{22}=\frac{1-v_{13}v_{31}}{E_{1}E_{3}\Gamma }, & C_{33}=\frac{1-v_{12}v_{21}}{E_{1}E_{2}\Gamma } \end{array}\\ \begin{array}{cc} C_{44}=G_{23},\; & \;C_{55}=G_{31} \end{array}\\ \begin{array}{c} C_{66}=G_{12} \end{array}\\ \Gamma =\frac{1-v_{12}v_{21}-v_{23}v_{32}-v_{13}v_{31}-2v_{12}v_{23}v_{31}}{E_{1}E_{2}E_{3}} \end{array}\right.$$

Under simulated real-world loading conditions, the Tsai–Wu tensor failure criterion is applied to evaluate the material stress state and predict potential failure [29]. When the stress at a specific point satisfies the failure criterion, that point is classified as failed. As a result, its elastic constants are degraded, which modifies the corresponding stiffness matrix elements and generates an updated post-damage material stiffness matrix. For material points that enter the damage evolution phase, this updated post-damage stiffness matrix is used in subsequent load step calculations. In contrast, for points that do not meet the failure criterion, the original stiffness matrix remains unchanged. This iterative process of stiffness matrix updating continues until structural failure is reached.

## 3. Numerical Simulation Under Medium Strain Rate Loading Conditions

### 3.1. Finite Element Model

**Figure 3:**
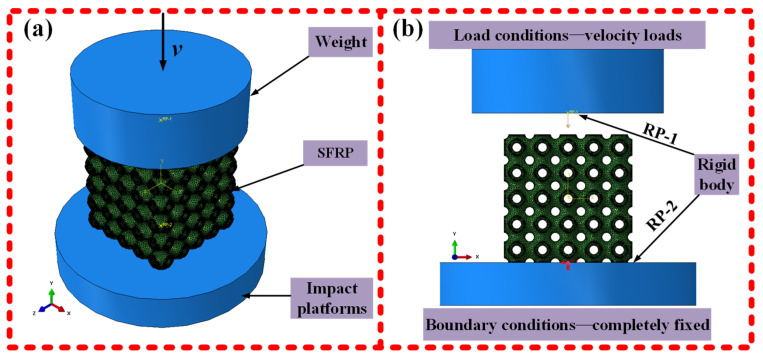
Numerical modelling of SFRP porous structures at medium strain rates. (**a**) Overall model: SFRP sandwiched between upper (velocity *v*) and lower rigid bodies; (**b**) Boundary/loading: upper (RP-1, velocity load); lower (RP-2, fully constrained).

The numerical model of the SFRP porous structure under medium strain rate conditions was established, as shown in Figure 3. the strength of the drop weight is significantly higher than that of the SFRP porous structure. Moreover, the stiffness of both the drop weight and the bottom platform under actual working conditions is considerably greater than that of the SFRP porous structure. Therefore, the top and bottom cylinders are modeled as rigid bodies in the numerical simulation. To simulate the stationary impact platform encountered in real-world applications, the bottom cylinder is fully constrained as part of the boundary conditions. The loading condition is applied by assigning an initial velocity to the upper rigid cylinder at the beginning of the simulation, which replicates the initial kinetic energy of the falling weight during an actual impact event [30].

### 3.2. Material Model Parameters

The equivalent elastic constants and material strength parameters for the fine-scale single-cell model were directly obtained from the literature [31], as presented in Table 2 and Table 3 below. These parameters were then assigned to the structural units of the macroscopic model, allowing for the analysis of the structural response at the macro-scale.

The elastic modulus of the macroscopic model for short fiber-reinforced composites can be determined using the Halpin–Tsai equation modified by Shokrieh [32], which is formulated as follows:

(5) $$\left\{\begin{array}{c} E_{c}=aE_{11}+\left(1-a\right)E_{22}\\ a=0.13+0.0815V_{f}-1.669\left(\frac{E_{m}}{E_{f}}\right) \end{array}\right.$$

The numerically simulated material parameters and strength values used for the macroscopic model of short fiber-reinforced composites are shown in Table 4 [33,34], and were numerically simulated using the Tsai–Wu tensor failure criterion as well as the stiffness degradation model.

## 4. Experimental Results and Analysis

### 4.1. Compressive Stress–Strain Relationship

The charge type accelerometer has a sensitivity *S* of 12.12 pC/(m/s^−2^) and generates a charge when it is subjected to acceleration, and the relationship between the charge *Q* and the acceleration *a* can be expressed as [35,36,37]

(6) Q=S×a

The role of the charge amplifier is to convert the charge signal into a voltage signal, with the gain *G* set to 0.1 V/pC. The resulting voltage signal *V* is then captured by the oscilloscope. It reads

(7) V=Q×G

The acceleration expression can be derived from the associated Equations (6) and (7) as follows:

(8) $$a=\frac{V}{S\times G}$$

The velocity *v* is obtained by integrating the acceleration *a* over the time *t*. The displacement *x* is obtained by integrating the velocity *v* over the time *t* using the same numerical integration method, and the strain is then calculated as [38,39]

(9) $$\varepsilon =\frac{\Delta x}{h_{0}}$$

where ∆*x* is the displacement change and *h*_0_ is the original height of the specimen.

The mass of the falling hammer *m* is 7.19 kg, and the cross-sectional area of the specimen is *A*_0_. According to Newton’s second law, the stress exerted by the falling hammer on the specimen can be expressed as

(10) $$\sigma =\frac{ma}{A_{0}}$$

**Figure 4:**
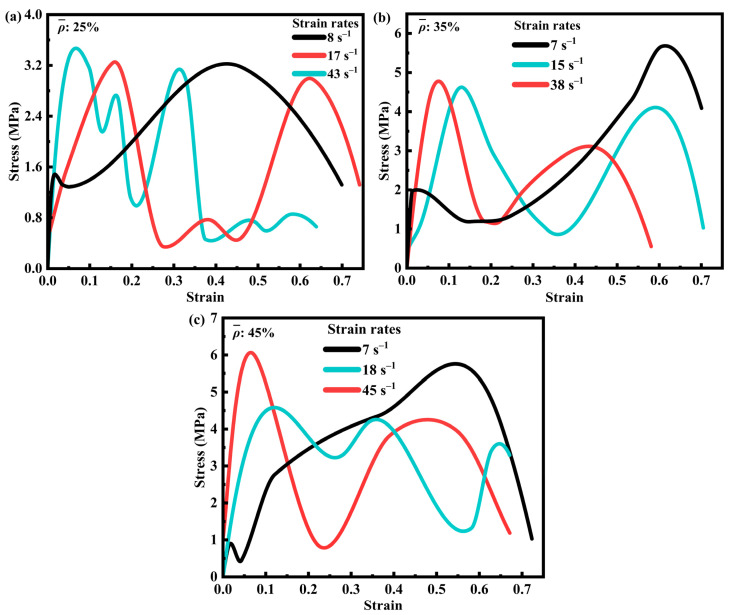
Stress–strain curves of SFRP porous structures with three volume fractions at different strain rates. (**a**) Stress-strain curves of SFRP porous structure with 25% volume fraction at strain rates of 8 s^−1^, 17 s^−1^, and 43 s^−1^; (**b**) Stress-strain curves of SFRP porous structure with 35% volume fraction at strain rates of 7 s^−1^, 15 s^−1^, and 38 s^−1^; (**c**) Stress-strain curves of SFRP porous structure with 45% volume fraction at strain rates of 7 s^−1^, 18 s^−1^, and 45 s^−1^.

At the conclusion of the impact experiment, time–voltage signals recorded by the oscilloscope were processed to obtain the stress–strain curves of the SFRP porous structure. Figure 4 illustrates these curves for three different volume fractions of SFRP porous structures under varying strain rates. The curves display multiple peaks, primarily due to localized stresses generated during the layer-by-layer fragmentation of the specimen. Image analysis of the impact process reveals that the drop hammer experienced slight rebound after the initial impact, momentarily losing contact with the specimen and causing intermittent loading cycles. These fluctuations in the curves result from the combined effects of external loading interruptions and progressive material failure. In Figure 4a, the stress–strain curves of the 25% volume fraction SFRP porous structure show a clear trend: peak stress increases with the strain rate, particularly within the range of 8 s^−1^ to 43 s^−1^. At higher strain rates, the internal strain rate strengthening mechanism becomes predominant, leading to enhanced structural strength and a steeper ascending portion of the curve. This behavior indicates a significant strengthening effect, where peak stress occurs at progressively smaller strain values, following the principle that higher strain rates induce more pronounced strengthening. Because at this fraction, characterized by low porosity (unit-cell wall thickness: 0.438 mm) and uniform fiber dispersion with relatively stable interfacial bonding, strain rate hardening mechanisms (e.g., matrix plastic hardening and enhanced fiber–matrix co-loading capacity) dominate the mechanical response as the strain rate increases from 8 s^−1^ to 43 s^−1^. Insufficient time exists for significant interfacial debonding or fiber clustering to occur. Consequently, the stress curve exhibits a steep ascending slope and a reduced peak strain (Figure 4a), indicating that the material readily enters an elastic strengthening phase at high strain rates, with deformation concentrated in global compression rather than localized damage.

Figure 4b presents the stress–strain curves for the 35% volume fraction SFRP porous structure, which exhibits higher porosity and relative fiber content. These characteristics promote more uniform stress transfer at the fiber–matrix interface. At lower strain rates, the combined effects of strain rate strengthening and interfacial synergy lead to higher peak stresses. However, under medium strain rate loading, stress concentrations at interfacial defects and thermal softening between the fibers and matrix counteract the strain rate strengthening effect, resulting in the lowest peak stress. At higher strain rates, the strain rate strengthening mechanism becomes dominant, as the loading time is insufficient to allow significant damage evolution, thereby producing peak stresses that exceed those observed at medium strain rates. Because specimens with a 35% volume fraction have thicker unit-cell walls (0.613 mm), the increased fiber density exacerbates local interfacial stress concentrations. At the medium strain rate (15 s^−1^), the rate of impact energy input balances the damage evolution rate: strain rate hardening enhances the load-bearing capacity, while concurrent interfacial debonding and fiber–matrix slip cause multi-peak fluctuations in the stress curve (Figure 4b). Conversely, at the high strain rate (38 s^−1^, impact duration ~0.015 s), damage lacks sufficient time to propagate, allowing strain rate hardening to dominate again and resulting in a peak stress exceeding that at the medium strain rate.

Figure 4c displays the curves for the 45% volume fraction SFRP porous structure, which has lower porosity compared to the previous two configurations. At lower strain rates, fiber bridging delays crack propagation and, together with interfacial synergy, contributes to elevated peak stresses. Under medium strain rate loading, the interaction among strain rate strengthening, damage evolution, and thermal softening results in reduced peak stresses, a behavior similar to that observed in the 35% volume fraction structure. At higher strain rates, the limited loading duration restricts substantial damage accumulation, enabling the strain rate strengthening effect to dominate and generate peak stresses greater than those at medium strain rates. Specimens with a 45% volume fraction exhibit a high fiber density (unit-cell wall thickness: 0.787 mm) and significant fiber clustering, leading to pronounced interfacial heterogeneity. At the medium strain rate (18 s^−1^), the detrimental effects of damage evolution (e.g., fiber fracture and matrix brittle cracking) outweigh strain rate hardening, minimizing the peak stress. At a high strain rate (45 s^−1^), the material maintains higher integrity due to the “inertial effect”, allowing the instantaneous load-bearing capacity of the fiber network to manifest and leading to a recovery of peak stress (Figure 4c). Furthermore, at low strain rates (7 s^−1^), the gentle stress rise observed for both the 35% and 45% volume fractions reflects ample time for damage accumulation, allowing interfacial defects to propagate gradually rather than causing sudden failure.

In summary, the 25% volume fraction SFRP porous structure exhibits a dominant strain rate strengthening effect, with peak stress consistently increasing as the strain rate increases, demonstrating a straightforward mechanical response. In contrast, for the 35% and 45% volume fraction structures, the interplay between strain rate strengthening, damage evolution, and thermal softening results in more complex stress–strain profiles. These materials display the lowest peak stress at medium strain rates and the highest peak stress at higher strain rates, which are attributed to the superior dominance of strain rate strengthening mechanisms under such conditions. Furthermore, at lower to medium strain rates, the material experiences slower plastic deformation and damage accumulation, leading to a more gradual increase in stress compared to higher strain rates. Otherwise, the initial segment (strain < 5%) of the black curve at a low strain rate (7 s^−1^) has a steeper slope, which essentially reflects that the material has a higher initial elastic modulus at low strain rates. At the initial stage of impact loading (strain < 5%), the material is in the elastic deformation stage, where stress is mainly transmitted through the fiber–matrix interface, and the integrity of the interface directly determines the initial stiffness (slope of the curve). At a low strain rate (7 s^−1^), the loading speed is slow (the impact duration is about 15–25 ms), allowing sufficient time for the fibers and matrix to achieve uniform stress transmission. Through mesoscopic observations, it is found that no debonding occurs at the fiber–matrix interface during this stage, and the fibers and matrix bear the load synergistically, resulting in a higher overall equivalent elastic modulus and hence a steeper slope of the initial segment of the curve. At higher strain rates (e.g., 17–43 s^−1^), the loading speed is fast (duration < 15 ms), causing stress to accumulate rapidly at the interface. Partial micro-debonding initiates prematurely at weaker interfaces, leading to a decrease in stress transmission efficiency. At this point, the matrix enters the plastic stage in advance due to rapid deformation, the equivalent elastic modulus decreases, and the slope of the initial segment of the curve becomes gentler.

**Figure 5:**
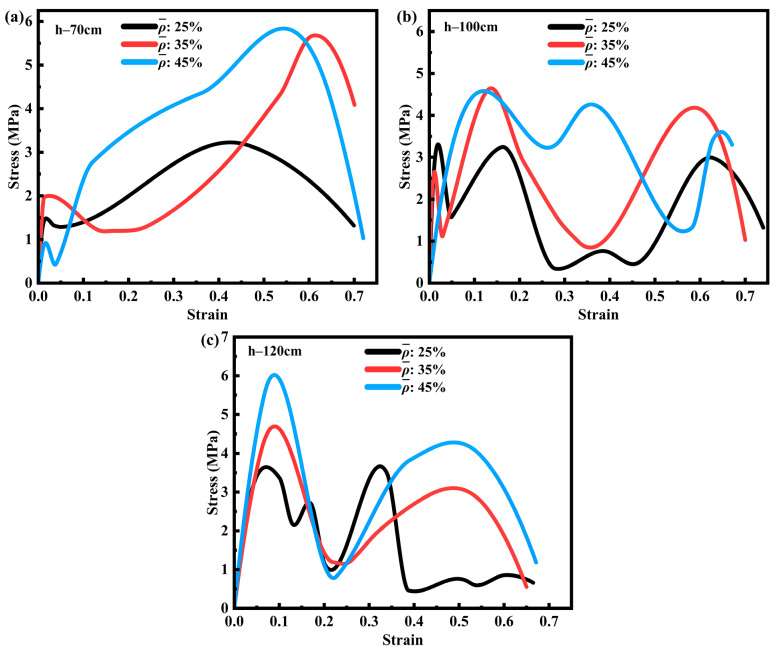
Stress–strain curves of SFRP porous structures at varying volume fractions and drop-weight heights. (**a**) Stress-strain curves of SFRP porous structures with 25%, 35%, and 45% volume fractions at a drop height of 70 cm; (**b**) Stress-strain curves of SFRP porous structures with 25%, 35%, and 45% volume fractions at a drop height of 100 cm; (**c**) Stress-strain curves of SFRP porous structures with 25%, 35%, and 45% volume fractions at a drop height of 120 cm.

The impact energy introduced into the system is primarily determined by the drop height. To systematically examine its influence on the stress–strain behavior of SFRP porous structures, a comprehensive analysis was carried out on the stress–strain curves of specimens with three distinct volume fractions, each subjected to impacts at three different drop heights. The findings of this study are summarized in Figure 5. The analysis indicates that at a given drop height, an increase in the volume fraction corresponds to a higher peak stress. Moreover, when the drop height is increased from 70 cm to 120 cm, the peak stress of SFRP structures across all three volume fractions demonstrates a general upward trend. Furthermore, notable variations are observed in the stress–strain curves among different volume fractions: specimens with a 45% volume fraction exhibit more pronounced stress fluctuations at elevated drop heights, whereas the peak stress and amplitude of fluctuation for those with 25% and 35% volume fractions display distinct curve characteristics as the drop height increases.

### 4.2. Energy Absorption Characteristics

**Figure 6:**
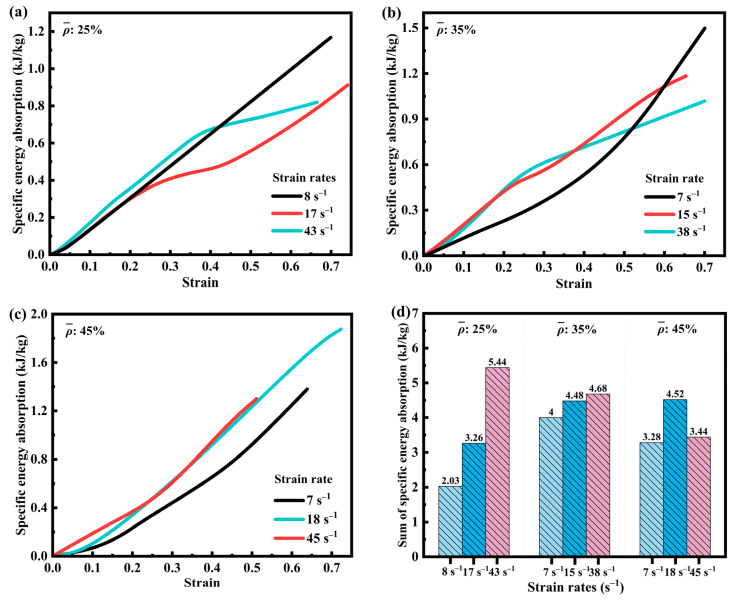
SEA of SFRP structures with three volume fractions at different medium strain rates. (**a**) SEA-strain curves of SFRP porous structure with 25% volume fraction at strain rates of 8 s^−1^, 17 s^−1^, and 43 s^−1^; (**b**) SEA-strain curves of SFRP porous structure with 35% volume fraction at strain rates of 7 s^−1^, 15 s^−1^, and 38 s^−1^; (**c**) SEA-strain curves of SFRP porous structure with 45% volume fraction at strain rates of 7 s^−1^, 18 s^−1^, and 45 s^−1^; (**d**) Comparison of the sums of SEA of three volume fraction SFRP porous structures at different strain rates.

Figure 6 illustrates the variation of specific energy absorption (SEA) [40,41] with strain for three volume fractions of SFRP porous structures at different medium strain rates. As can be observed, all SEA–strain curves exhibit a continuous increasing trend with increasing strain. Figure 6a indicates that the SFRP porous structure with a 25% volume fraction contains relatively fewer interfacial defects, which is attributed to its lower volume fraction. Consequently, its mechanical behavior is predominantly governed by the strain rate strengthening mechanism. At higher strain rates, the improved synergistic load-bearing capacity at the fiber–matrix interface significantly enhances the SEA during deformation. This efficiency stems from stable energy dissipation paths enabled by low porosity and uniform interfaces. Increased strain rates enhance the co-deformation capability of fibers and the matrix, improve plastic work conversion efficiency, and promote uniform shear band propagation, preventing premature localized failure from interrupting energy absorption. As shown in Figure 6d, the SEA at a strain rate of 43 s^−1^ is 2.18 kJ/kg higher than that at 17 s^−1^ and 3.41 kJ/kg higher than that at 8 s^−1^, highlighting the significant impact of strain rate strengthening on materials with low volume fractions.

In Figure 6b, the SFRP porous structure with a 35% volume fraction demonstrates a slower increase in SEA at the lower strain rate (7 s^−1^) during the initial loading phase. The slope of the curve becomes steeper in the subsequent stage, indicating that the material absorbs energy in a stable and continuous manner. At higher strain rates, the curves exhibit a sharp initial rise followed by a gradual plateau. This behavior reflects the predominance of the strain rate strengthening mechanism in the early deformation stage, while the progressive evolution of interfacial damage gradually diminishes the material’s capacity for energy absorption in the later stage.

Figure 6c presents the specific energy absorption (SEA) behavior of the SFRP porous structure with a 45% volume fraction. At the lower strain rate (7 s^−1^), the curve exhibits characteristics similar to those of the 35% volume fraction structure, featuring a gradual increase during the initial deformation stage followed by an accelerated rise. At higher strain rates (18 s^−1^, 43 s^−1^), the strain rate strengthening mechanism predominates in the early phase, leading to a steeper slope compared to that at 7 s^−1^. In the subsequent stage, as the evolution of interfacial damage increasingly counteracts the strain rate strengthening effect, the slope diminishes. However, due to the residual structural load-bearing capacity, pore compression, and continuous material deformation, the curve sustains a steady upward trend.

Figure 6d summarizes the specific energy absorption (SEA) behavior of SFRP porous structures with three different volume fractions at varying strain rates. For the structure with a 25% volume fraction, the SEA consistently increases with the strain rate, consistent with the characteristic response of strain rate dominance observed in low-volume fraction materials. In the case of the 35% volume fraction structure, the SEA at 38 s^−1^ is slightly higher than that at 15 s^−1^ by 0.2 kJ/kg and 0.68 kJ/kg greater than that at 7 s^−1^, indicating stable energy absorption performance at elevated strain rates. For the 45% volume fraction structure, SEA at 18 s^−1^ is 2.18 kJ/kg higher than at 8 s^−1^ and 1.24 kJ/kg higher than at 7 s^−1^, yet slightly lower than at 45 s^−1^. This behavior suggests that at 18 s^−1^, a dynamic equilibrium is achieved between strain rate strengthening and interfacial damage evolution, resulting in an optimal energy absorption capacity.

Overall, for low-volume fraction SFRP porous structures (25%), the SEA increases consistently with strain rate, underscoring the predominant influence of strain rate strengthening. For medium-volume and high-volume fraction structures (35% and 45%), the interaction between strain rate strengthening and damage evolution introduces a higher degree of complexity, leading to noticeable variations in SEA across different strain rates. This phenomenon reflects the complex dynamic mechanical response exhibited by medium- and high-volume fraction SFRP porous structures.

**Figure 7:**
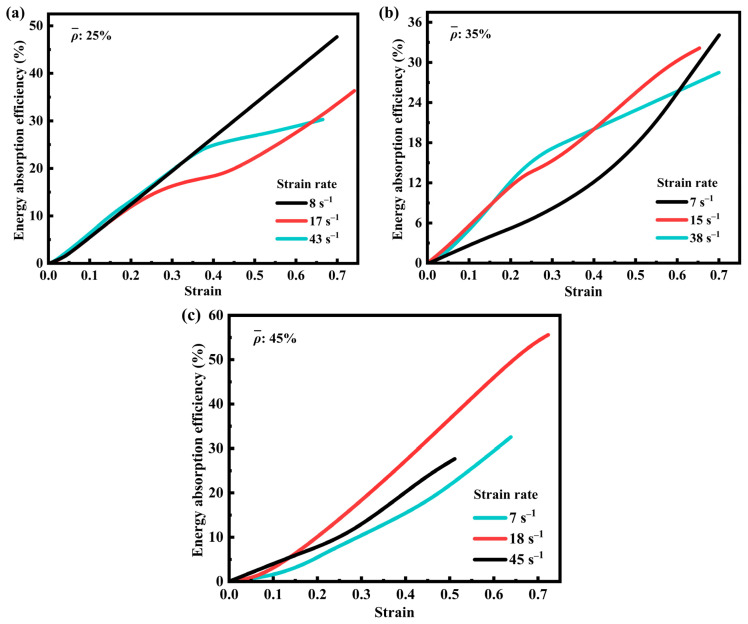
The EAE of SFRP structures with three volume fractions at different medium strain rates. (**a**) EAE-strain curves of SFRP porous structure with 25% volume fraction at strain rates of 8 s^−1^, 17 s^−1^, and 43 s^−1^; (**b**) EAE-strain curves of SFRP porous structure with 35% volume fraction at strain rates of 7 s^−1^, 15 s^−1^, and 38 s^−1^; (**c**) EAE-strain curves of SFRP porous structure with 45% volume fraction at strain rates of 7 s^−1^, 18 s^−1^, and 45 s^−1^.

To address the limitations associated with relying exclusively on specific energy absorption metrics, the variation of energy absorption efficiency (EAE) with strain was investigated for SFRP porous structures with three volume fractions at various medium strain rates, as illustrated in Figure 7 [42,43]. As depicted in Figure 7a, the SFRP porous structure with a 25% volume fraction demonstrates a steady increase in EAE at a lower strain rate (8 s^−1^), indicating a stable response to progressive strain accumulation. At higher strain rates (14 s^−1^ and 43 s^−1^), the curves exhibit a steep initial rise followed by a more gradual increase during the later deformation stages. This behavior arises from the combined influence of strain rate reinforcement and constraints imposed by deformation limits. The steep initial slope of the energy absorption efficiency curve at high strain rates (Figure 7a) reflects the “gain effect” of strain rate hardening concentrated in the early deformation phase.

In Figure 7b, for the 35% volume fraction at 7 s^−1^, the energy absorption efficiency remains relatively flat until ε ≈ 0.4, as slowly accumulating damage allows the matrix to continuously absorb energy through plastic folding. At 15 s^−1^ and 38 s^−1^, initial strain rate hardening drives rapid efficiency increases, but subsequent interfacial debonding reduces fiber load-bearing capacity, causing the efficiency growth rate to slow (Figure 7b). This fluctuation corresponds to the multi-peak stress–strain behavior, indicating alternating active and quiescent phases in damage evolution.

Figure 7c illustrates the EAE behavior of the 45% volume fraction SFRP porous structure. At a lower strain rate (7 s^−1^), the mechanism is similar to that of the 35% volume fraction structure, characterized by slower damage evolution and extended energy absorption accumulation periods. At a strain rate of 18 s^−1^, the curve shows a consistent upward trend and reaches the maximum EAE value, indicating that strain rate reinforcement prevails over interfacial defect evolution. At a higher strain rate (45 s^−1^), the curve rises rapidly during the early deformation stage but grows more gradually thereafter, reflecting a transition from strain rate strengthening dominance to constraints imposed by interfacial defect damage. The 45% volume fraction exhibits the peak energy absorption efficiency at 18 s^−1^, where strain rate hardening partially counteracts damage effects: the dense fiber network provides high initial load-bearing, while energy is dissipated through matrix plastic drawing (fibrillated structures) and pore compression. However, at 45 s^−1^, the proportion of brittle fracture increases, reducing the residual structure’s energy absorption capacity and flattening the efficiency curve later on, confirming the “high-strength, low-ductility” trade-off.

Overall, the energy absorption mechanisms in SFRP porous structures are influenced by both the volume fraction and strain rate, primarily due to variations in the interfacial defect density. Structures with low volume fractions are predominantly governed by strain rate strengthening, resulting in a relatively simple dynamic mechanical response. In contrast, medium- and high-volume fraction structures contain a greater number of interfacial defects, leading to a more complex interaction between strain rate enhancement and the evolution of interfacial defect damage. This complexity is evident in the morphological characteristics of the strain–EAE curves.

### 4.3. Damage and Failure Modes

**Figure 8:**
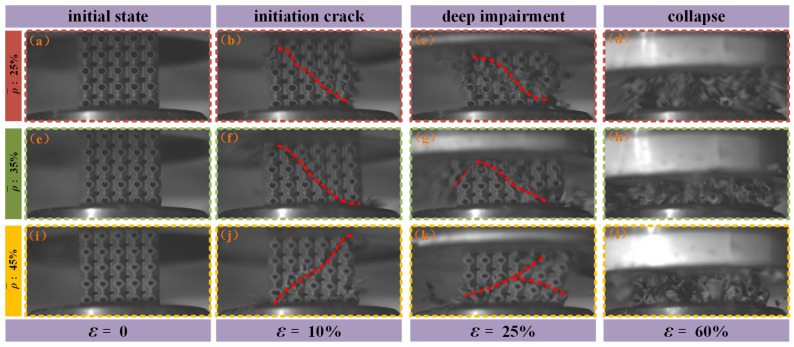
Damage and failure modes of SFRP structures with three volume fractions at various strains. (**a**) 25% volume fraction, ε = 0 (initial state); (**b**) 25% volume fraction, ε = 10% (initiation crack); (**c**) 25% volume fraction, ε = 25% (deep impairment); (**d**) 25% volume fraction, ε = 60% (collapse); (**e**) 35% volume fraction, ε = 0 (initial state); (**f**) 35% volume fraction, ε = 10% (initiation crack); (**g**) 35% volume fraction, ε = 25% (deep impairment) (**h**) 35% volume fraction, ε = 60% (collapse); (**i**) 45% volume fraction, ε = 0 (initial state); (**j**) 45% volume fraction, ε = 10% (initiation crack); (**k**) 45% volume fraction, ε = 25% (deep impairment) (**l**) 45% volume fraction, ε = 60% (collapse).

The stress–strain curves obtained from drop-weight impact tests on SFRP porous structures at medium strain rates indicate that the overall structural response during loading evolves through several distinct phases: elastic deformation, plastic deformation, shear damage, extensive damage, compressive collapse, and densification rebound. The damage and failure modes observed in SFRP porous structures with three different volume fractions at various strain levels (0%, 10%, 25%, and 60%) under impact loading conditions are presented in Figure 8. At a compressive strain of approximately ε = 10%, a 45° inclined crack forms in structures with all three volume fractions. This crack generally propagates from the top layer to the bottom layers, forming the initial shear zone (as illustrated in Figure 8b,f,j). This phase represents the onset of localized damage induced by impact loading.

As the compressive strain increases to ε = 25%, the high impact energy induces rapid crack propagation along the weakest stress transmission paths. Multiple cracks initiate and propagate in various regions of the structure, intersecting and coalescing, thereby resulting in a continuous expansion of the damaged zone. At this stage, the structure transitions into the extensive damage phase, and its load-bearing capacity undergoes a substantial reduction (as illustrated in Figure 8c,g,k).

When the compressive strain reaches ε = 60%, the extensively cracked regions of the structure are no longer capable of withstanding the applied impact forces. This leads to extensive structural collapse, resulting in the formation of fragments of varying sizes. These fragments are ejected from the structure due to the release of accumulated internal stresses and the continued action of external impact forces (as illustrated in Figure 8d,h,l). At this stage, the structural integrity is entirely compromised, and the structure is rendered completely destroyed.

**Figure 9:**
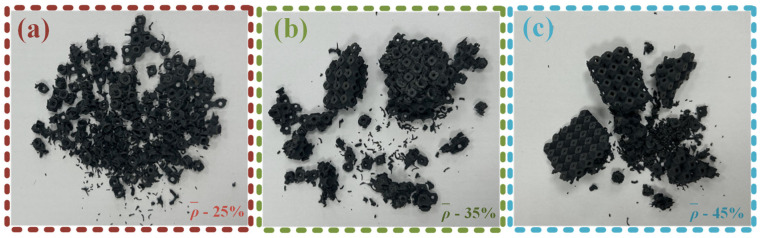
Morphology of damaged fragments after the failure of SFRP structures with three volume fractions. (**a**) Fragment morphology of SFRP porous structure with 25% volume fraction; (**b**) Fragment morphology of SFRP porous structure with 35% volume fraction; (**c**) Fragment morphology of SFRP porous structure with 45% volume fraction.

At the end of the experiment, the fractured fragments of SFRP porous structures with three different volume fractions were examined and compared, as illustrated in Figure 9. The analysis reveals distinct differences in post-failure morphology among the structures based on their volume fraction. For the SFRP porous structure with a 25% volume fraction, the failure mode is characterized by complete fragmentation, leaving no identifiable residual structural form. In contrast, the structures with 35% and 45% volume fractions exhibit larger, block-like residual fragments, indicating greater structural retention after compression-induced failure. Notably, the SFRP porous structure with a 45% volume fraction demonstrates superior post-impact integrity, maintaining an intact single-cell layered structure despite the applied impact loading. These findings indicate that SFRP porous structures with higher volume fractions exhibit an enhanced load-bearing capacity under impact loading conditions. The increased material density contributes to improved structural robustness and damage resistance, as evidenced by the preserved residual structures observed in the higher volume fraction specimens.

**Figure 10:**
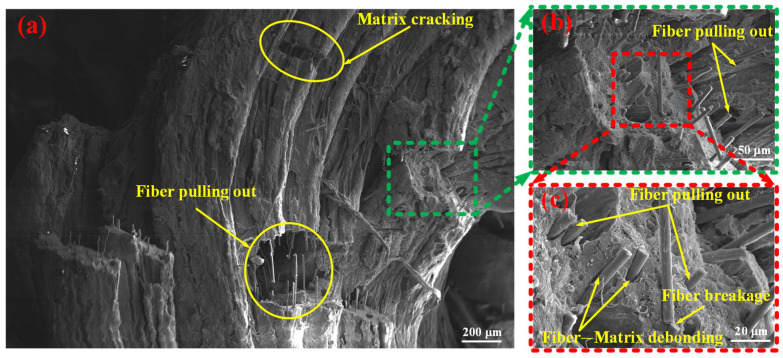

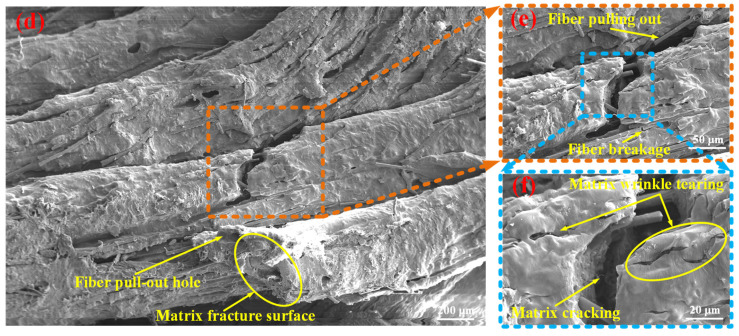
Mesoscopic damage of the SFRP porous structure with a 25% volume fraction at a medium strain rate. (**a**) Low-magnification SEM image: overall fracture surface showing fiber pulling out and matrix cracking as primary damage features; (**b**) Magnified view (green-dashed region in (**a**)): detailed observation of extensive fiber pulling out behavior; (**c**) Higher-magnification view (red-dashed region in (**b**)): composite damage mechanisms revealed: fiber pulling out, fiber breakage, and fiber–matrix debonding at the interface; (**d**) Low-magnification SEM image: fracture surface with a visible fiber pull-out hole and rough matrix fracture surface; (**e**) Magnified view (orange-dashed region in (**d**)): localized fiber pulling out and fiber breakage; (**f**) Higher-magnification view (blue-dashed region in (**e**)): matrix-dominated damage: matrix wrinkle tearing and matrix cracking.

Scanning electron microscopy (SEM) observations were performed on the fractured fragments of the SFRP porous structures following the experiments, offering valuable insights into the mesoscopic damage characteristics. Figure 10 presents the mesoscopic damage features of the SFRP porous structure with a 25% volume fraction under medium strain rate loading conditions. The observations reveal several key aspects related to the fiber–matrix interaction and associated damage mechanisms. The interfacial bonding quality between the fibers and matrix is found to be weak, with widespread debonding observed throughout the structure. Fiber pull-out is extensively present, with some fibers displaying fracture patterns consistent with plastic shear failure. In regions where fiber pull-out has occurred, residual voids or pull-out channels are clearly visible on the matrix surface. The matrix in these fiber pull-out zones exhibits pronounced plastic deformation, characterized by surface folding and tearing, which contributes to the formation of an interconnected microcrack network. Additionally, distinct macroscopic cracks are identified within the matrix, and fragmented fibers are distributed across certain areas of the fracture surface. Collectively, these features—fiber pull-out, matrix deformation, and crack propagation—demonstrate the complex mechanical interactions between the fibers and matrix under impact loading, particularly in SFRP porous structures with lower volume fractions. This “progressive debonding” facilitates slow shear band propagation (Figure 8b–d), culminating in complete structural fragmentation (Figure 9a), with energy dissipated via fiber–matrix friction and matrix plastic deformation.

**Figure 11:**
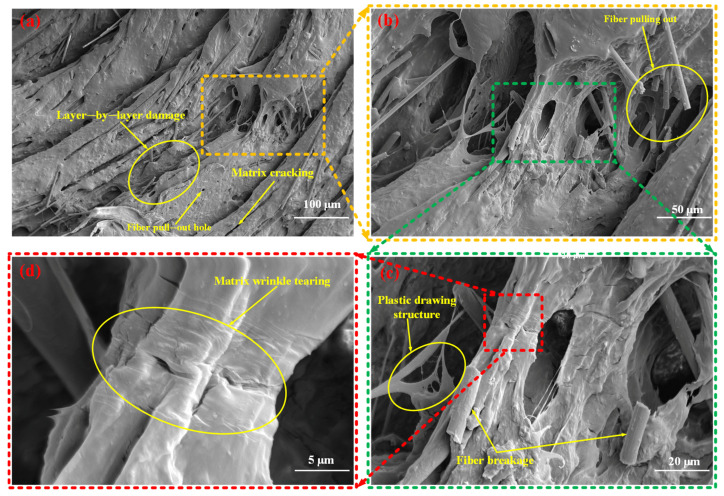
Mesoscopic damage of the SFRP porous structure with a 35% volume fraction at a medium strain rate. (**a**) Low-magnification SEM image: overall damage morphology featuring layer-by-layer damage, matrix cracking, and distinct fiber pull-out holes, illustrating the macroscopic damage distribution; (**b**) Magnified view (yellow-dashed region in (**a**)): detailed observation of fiber pulling out behavior and internal porous damage features; (**c**) Higher-magnification view (green-dashed region in (**b**)): microscale damage mechanisms revealed, including plastic drawing structures (matrix plastic flow characteristic) and fiber breakage; (**d**) High-magnification SEM image(red-dashed region in (**c**)): characteristic matrix wrinkle tearing morphology, reflecting localized shear deformation of the matrix.

Figure 11 presents the mesoscopic damage characteristics of the SFRP porous structure with a 35% volume fraction under medium strain rate loading conditions. The observations indicate that the structure exhibits fiber pull-out features, with fractured fibers evident on the matrix surface. In certain regions, a step-like laminated morphology is identified, suggesting progressive, layer-by-layer damage evolution. Additionally, the matrix shows folded textures and cracks induced by tearing, which reflect localized deformation behavior. Notably, a filamentary structure is observed in specific areas of the matrix, which can be attributed to the localized tensile flow of the matrix material caused by impact loading. This filamentary morphology illustrates the presence of plastic ductility under medium strain rate conditions. The matrix undergoes plastic flow, forming fibrillated structures, indicating retained ductility at medium strain rates. Fiber bridging delays crack penetration, leaving residual structures in blocky fragments (Figure 9b).

**Figure 12:**
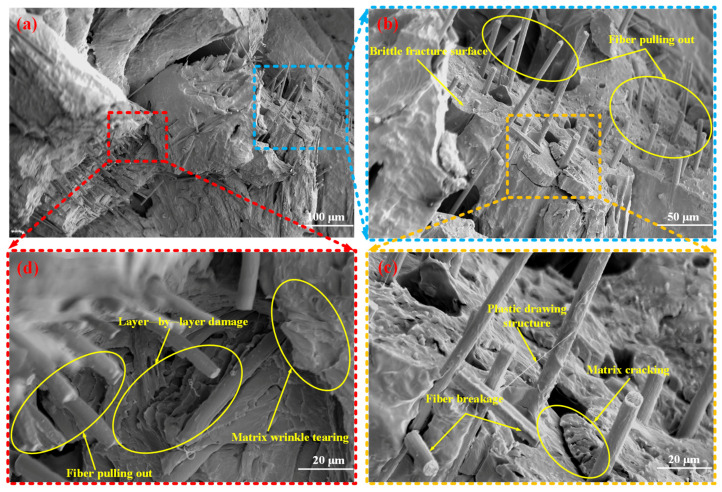
Mesoscopic damage of the SFRP porous structure with a 45% volume fraction at a medium strain rate. (**a**) Low-magnification SEM image: overall fracture morphology featuring brittle fracture surface and fiber pull-out; (**b**) Magnified view (blue-dashed region in (**a**)): detailed observation of brittle fracture surface and prominent fiber pull-out; (**c**) Higher-magnification view (yellow-dashed region in (**b**)): microscale damage mechanisms revealed, including plastic drawing structures, fiber breakage, and matrix cracking; (**d**) Magnified view (red-dashed region in (**a**)): characteristic damage features including layer-by-layer damage, matrix wrinkle tearing (reflecting localized shear deformation), and fiber pull-out.

Figure 12 presents the mesoscopic damage characteristics of the SFRP porous structure with a 45% volume fraction under medium strain rate loading conditions. The observations highlight several distinctive damage features associated with fiber–matrix interactions and matrix failure mechanisms. Fiber pull-out phenomena are prominently present in the 45% volume fraction structure, frequently accompanied by fiber fractures. In contrast to the damage features observed in the 25% and 35% volume fraction structures, some fibers in the 45% sample are partially embedded within filamentary matrix structures on the outer surface. This unique feature indicates localized fiber encapsulation, which may influence stress distribution and contribute to altered damage behavior. The matrix in the 45% volume fraction structure exhibits pronounced fracture characteristics, including macroscopic cracks and flat fracture surfaces typical of localized brittle failure. Additionally, the matrix surface displays folded tearing patterns and indications of layer-by-layer failure, suggesting residual plastic deformation. These findings imply that the damage mechanisms in this structure involve a complex combination of brittle and ductile failure modes. This leads to rapid densification at high strain rates and higher residual structural integrity (Figure 9c), but the energy absorption efficiency is limited by the sudden nature of brittle fracture.

Overall, the damage mechanisms of SFRP porous structures under medium strain rate loading exhibit distinct variations with the volume fraction. The 25% volume fraction structure is primarily characterized by fiber pull-out, along with matrix folding and tearing. The 35% volume fraction structure demonstrates notable plastic ductile matrix damage, resulting in the formation of filamentary structures, coupled with layer-by-layer failure behavior. The 45% volume fraction structure displays a combination of localized brittle failure and residual plastic deformation characteristics.

## 5. Numerical Simulation Results and Analysis

### 5.1. Damage and Failure Modes

**Figure 13:**
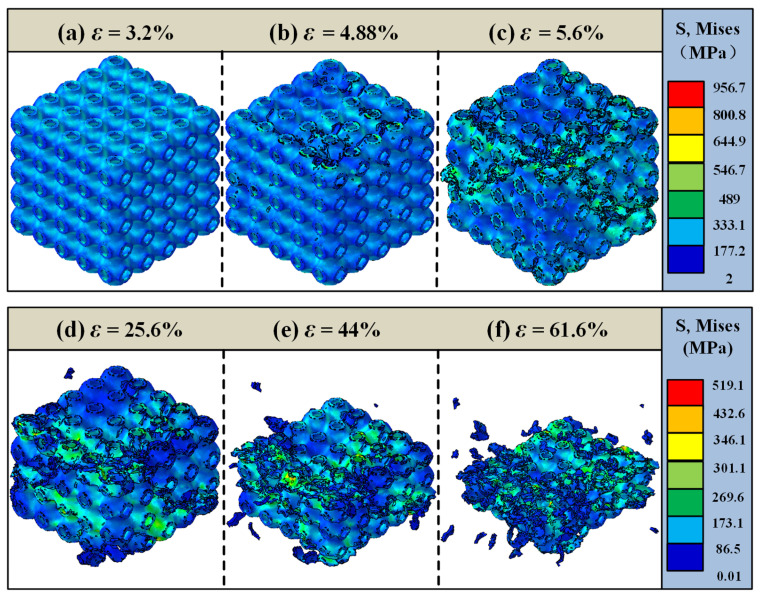
Failure analysis of the 25% volume fraction SFRP porous structure under medium strain rate loading. (**a**) ε = 3.2%, elastic deformation phase; (**b**) ε = 4.88%, initial crack sprouting; (**c**) ε = 5.6%, shear zone formation; (**d**) ε = 25.6%, aggravation of damage; (**e**) ε = 44%, collapse of the middle area; (**f**) ε = 61.6%, completely fails.

To clearly and comprehensively observe the failure evolution process of the structure, as well as to understand the damage initiation location, extension path, and final failure pattern, a 3D overall cloud diagram analysis was conducted. Figure 13 presents the overall failure analysis of the 25% volume fraction SFRP porous structure under medium strain rate loading. At a strain of ε = 3.2%, the cloud diagram reveals a uniform stress distribution across the structure, with no visible damage, indicating that the material remains in the elastic deformation stage. With continued loading at ε = 4.88%, small cracks begin to emerge around the pores in the upper-middle region of the structure, with trace defects observed in the single-cell tabular ring structure. As the damage progresses along the pore boundaries, the affected region expands further at ε = 5.6%, leading to shear damage in the upper and lower interlayer stress weak zones and forming an apparent shear zone. By ε = 25.6%, the damage intensifies, and a distinct shear zone extending from the upper left corner to the lower right corner is observed, with severe damage concentrated in the middle layer of the structure. Further loading to ε = 44% results in significant structural damage, with nearly two intermediate layers collapsing under compressive forces. Finally, at ε = 61.6%, the structure is fully compressed, nearly densified, and produces a substantial amount of fractured debris.

In summary, the 25% volume fraction SFRP porous structure under medium strain rate loading undergoes a progressive failure evolution process, transitioning from elastic deformation to damage initiation and propagation around the pore periphery, followed by plastic deformation dominance, and ultimately culminating in complete structural failure.

**Figure 14:**
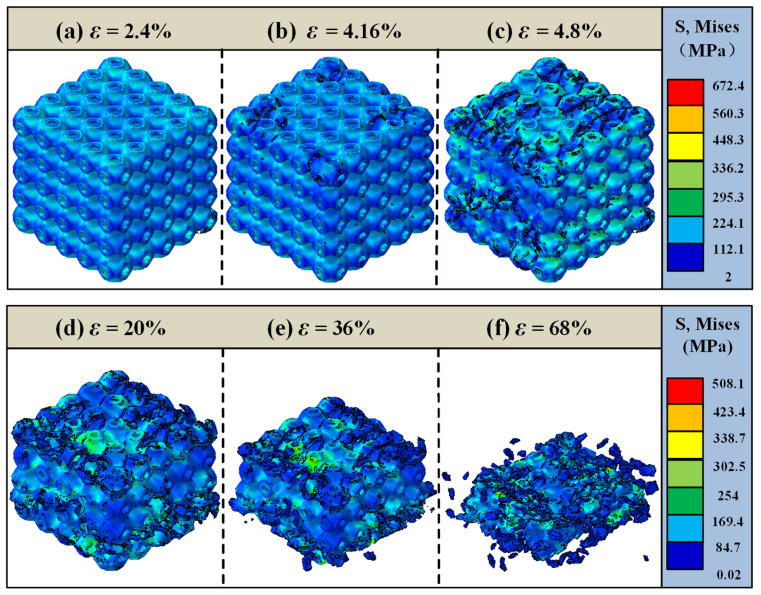
Failure analysis of the 35% volume fraction SFRP porous structure under medium strain rate loading. (**a**) ε = 2.4%, elastic deformation stage; (**b**) ε = 4.16%, slight damage appears in the middle region; (**c**) ε = 4.8%, damage propagates; (**d**) ε = 20%,shear damage is significant, forming a triangular shear band; (**e**) ε = 36%,the middle region compresses and collapses; (**f**) ε = 68%, structure completely loses its original form, becoming heavily densified with extensive material fragmentation.

Figure 14 presents the overall failure analysis of the 35% volume fraction SFRP porous structure under medium strain rate loading. At an initial strain of ε = 2.4%, the structure remains in the elastic deformation stage, demonstrating a uniform stress response. As the loading progresses, at ε = 4.16%, slight damage begins to appear, primarily concentrated in the middle region of the structure. With the continued input of impact energy, the damage gradually propagates, and by ε = 4.8%, multiple localized damage areas are observed. At a strain of ε = 20%, shear damage becomes increasingly pronounced, resulting in a distinct “triangular” shape of shear zones within the structure. As the strain reaches ε = 36%, the middle region undergoes compression and collapse, leading to a sharp reduction in the load-bearing capacity. Finally, at ε = 68%, the structure completely loses its original form, becoming heavily densified with extensive material fragmentation.

In comparison with the failure mode of the 25% volume fraction structure under medium strain rate loading, the 35% volume fraction structure demonstrates a more complex shear damage pattern and sustains a larger compressive strain before ultimate failure. These characteristics underscore the superior load-bearing capacity and enhanced structural performance of the 35% volume fraction structure under dynamic loading conditions.

**Figure 15:**
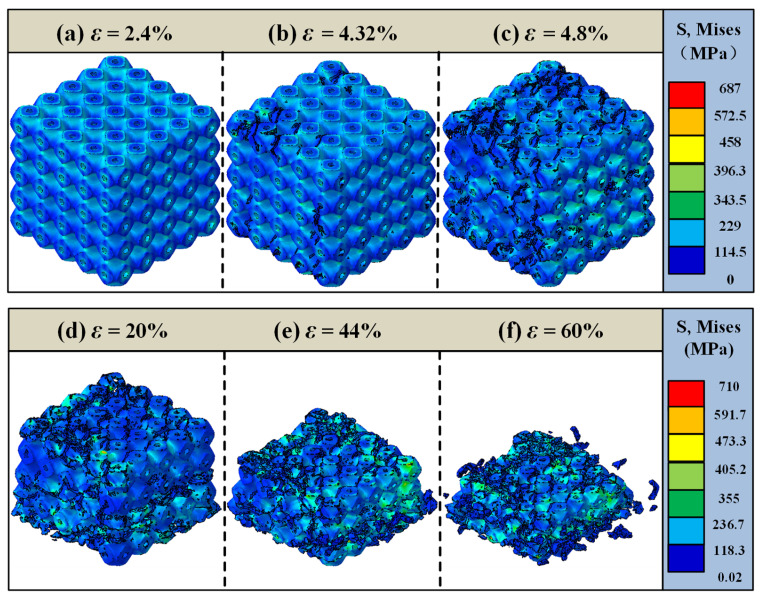
Failure analysis of the 45% volume fraction SFRP porous structure under medium strain rate loading. (**a**) ε = 2.4%, elastic deformation stage; (**b**) ε = 4.32%, initial cracks begin to develop along pore boundaries; (**c**) ε = 4.8%, cracks expand rapidly; (**d**) ε = 20%, irreversible compressive failure occurs in the middle layer of the structure; (**e**) ε = 44%, distinct shear damage appears; (**f**) ε = 60%, structure completely fails.

Figure 15 illustrates the overall failure analysis of the 45% volume fraction SFRP porous structure under medium strain rate loading. At an initial strain of ε = 2.4%, the structure exhibits minimal damage, remaining primarily within the elastic deformation stage. As the loading progresses to ε = 4.32%, initial cracks begin to develop along the pore boundaries, marking the transition to the elastic–plastic deformation stage. By ε = 4.8%, these cracks rapidly expand and propagate, leading to damage that is more widespread and diffusely distributed compared to the 35% volume fraction structure at the same compressive strain. When the strain reaches ε = 20%, irreversible compressive failure occurs in the middle layer of the structure. As the damage evolves further, at ε = 44%, distinct shear damage becomes apparent, and the structure approaches densification. Finally, at ε = 60%, the structure undergoes complete failure, losing its original load-bearing functionality.

In comparison with the 35% volume fraction SFRP porous structure, the 45% volume fraction structure demonstrates a higher maximum stress in the cloud diagram, indicative of an enhanced load-bearing capacity. However, its final failure strain is lower due to the denser fiber network within the higher volume fraction structure. This increased fiber density reinforces the load-bearing capability but diminishes deformation compatibility, as crack propagation is more likely to follow interfacial weaknesses. Consequently, the failure mode of the higher volume fraction structure under medium strain rate loading tends toward brittle fracture, characterized by a superior load-bearing capacity at the expense of a reduced deformation capacity.

Overall, numerical simulations reveal that shear bands initiate at approximately 45° within the strain range of ε = 5–20% across all volume fractions (Figure 13c, Figure 14d and Figure 15d). This phenomenon is attributed to the material’s principal stress direction (aligned with the loading axis) and shear strength anisotropy. Low volume fraction specimens exhibit concentrated, penetrating shear bands (Figure 13d), where the uniform stress distribution from dispersed fibers results in singular failure paths. Conversely, high volume fraction specimens develop diffuse shear bands (Figure 15d). Here, the fiber density intensifies stress concentrations, triggering multiple intersecting shear bands, consistent with experimental observations of multidirectional crack convergence (Figure 8k). The damage evolution of SFRP porous structures varies significantly with the volume fraction. Structures with lower volume fractions primarily exhibit localized shear-dominated damage, characterized by concentrated damage paths and less diffuse propagation. In contrast, high volume fraction structures display a multidirectional and more diffuse damage pattern, reflecting the influence of their denser fiber networks. Additionally, the 45% volume fraction structure demonstrates brittle damage characteristics, defined by high strength but low toughness. This behavior arises from the intensified competition between two failure mechanisms: fiber–matrix interface debonding and fiber fracture. The denser fiber network enhances the load-bearing capacity but compromises deformation compatibility, making cracks more likely to propagate along interfacial weaknesses and accelerating the onset of brittle fracture. The strain interval from damage initiation (ε = 4.32%) to complete failure (ε = 60%) for the 45% volume fraction is 55.68%, which is significantly smaller than the 58.4% interval (ε = 3.2% to 61.6%) for the 25% volume fraction. This demonstrates accelerated damage propagation at a higher fiber content, corroborating the experimental observation of faster crushing in 45% specimens at high strain rates and confirming the “high fiber density–stress concentration–accelerated damage” chain.

### 5.2. Comparison Between the Experimental Results and Numerical Simulations

**Figure 16:**
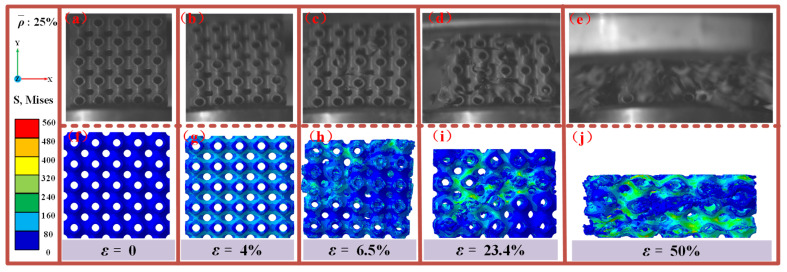
Comparison of the experimental observations and simulations of the damage failure modes of SFRP porous structures with a volume fraction of 25% at medium strain rates. (**a**) experimental observation at ε = 0; (**b**) experimental observation at ε = 4%; (**c**) experimental observation at ε = 6.5%; (**d**) experimental observation at ε = 23.4%; (**e**) experimental observation at ε = 50%; (**f**) stress cloud for damage failure modes at ε = 0; (**g**) stress cloud for damage failure modes at ε = 4%; (**h**) stress cloud for damage failure modes at ε = 6.5%; (**i**) stress cloud for damage failure modes at ε = 23.4%; (**j**) stress cloud for damage failure modes at ε = 50%.

Figure 16 presents a comparison between the experimental observations and numerical simulations of the damage and failure modes of an SFRP porous structure with a 25% volume fraction under medium strain rate loading. At ε = 0, the structure is in its initial state, with loading initiated thereafter. When the strain reaches ε = 4%, the experimental results indicate uniform deformation of the structure along the loading direction. Simultaneously, the stress cloud maps from the numerical simulation begin to reveal trace stress distributions, marking the elastic deformation stage. As loading progresses, experimental crack initiation at ε = 6.5% corresponds to simulated stress concentration at ε = 5.6% (Figure 12), with a minor discrepancy of 14%. This validates the model’s capture of stress concentration around pores (the weak points for crack initiation). This indicates the onset of crack damage initiation at specific regions of the structure. At a strain of ε = 23.4%, the experimental results reveal significant crack propagation and shear damage, consistent with the stress extension paths observed in the numerical simulation’s stress cloud diagrams. Damage develops rapidly at this stage, with shear zones becoming more pronounced. By ε = 50%, the experiment shows substantial compressive deformation of the structure, accompanied by a large damage region. This is mirrored in the numerical simulation, which accurately captures the progression of damage and the stress distribution.

Overall, the comparison demonstrates a strong correlation between experimental phenomena and numerical simulation results across all stages, including elastic deformation, crack initiation, shear damage propagation, and large-scale structural failure. The simulated stress cloud diagrams effectively replicate the stress distribution and damage evolution trends observed in the experiments, validating the numerical approach.

**Figure 17:**
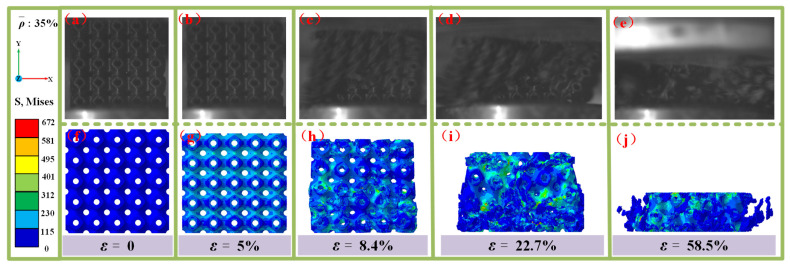
Comparison of experimental observations and simulations of the damage failure modes of SFRP porous structures with a volume fraction of 35% at medium strain rates. (**a**) experimental observation at ε = 0; (**b**) experimental observation at ε = 5%; (**c**) experimental observation at ε = 8.4%; (**d**) experimental observation at ε = 22.7%; (**e**) experimental observation at ε = 58.5%; (**f**) stress cloud for damage failure modes at ε = 0; (**g**) stress cloud for damage failure modes at ε = 5%; (**h**) stress cloud for damage failure modes at ε = 8.4%; (**i**) stress cloud for damage failure modes at ε = 22.7%; (**j**) stress cloud for damage failure modes at ε = 58.5%.

Figure 17 presents a comparison between the experimental observations and numerical simulations of the damage and failure modes of an SFRP porous structure with a 35% volume fraction subjected to medium strain rate loading. At ε = 0, the structure is in its initial unstressed state. When the strain reaches ε = 5%, the experimental results indicate minimal deformation, and the stress cloud diagram reveals a slight stress distribution. This marks the transition of the structure into the elastic deformation phase. As the strain increases to ε = 8.4%, damage cracks begin to form around the pores in the experiment, a phenomenon corroborated by localized stress concentrations observed in the numerical simulation. At ε = 22.7%, crack propagation intensifies, and a distinct shear zone emerges on the surface of the structure, oriented approximately 45° relative to the loading direction, verifying the model’s prediction of the interfacial damage evolution rate. The experiment and simulation both demonstrate shear damage progressing along this shear zone. By ε = 58.5%, the structure experiences substantial compressive deformation, accompanied by the generation of numerous discrete fragments, as captured in the stress cloud diagrams of the simulation.

In summary, the damage evolution of the 35% volume fraction SFRP porous structure follows a clear progression from elastic deformation to crack initiation, shear band formation, and eventual large-scale compression and fragmentation. Compared to the 25% volume fraction structure, the 35% volume fraction structure exhibits a faster rate of damage accumulation, progressing more rapidly from crack initiation to complete failure. This accelerated damage evolution is primarily attributed to the denser fiber network at higher porosity, which amplifies stress interactions and facilitates the development of localized damage.

**Figure 18:**
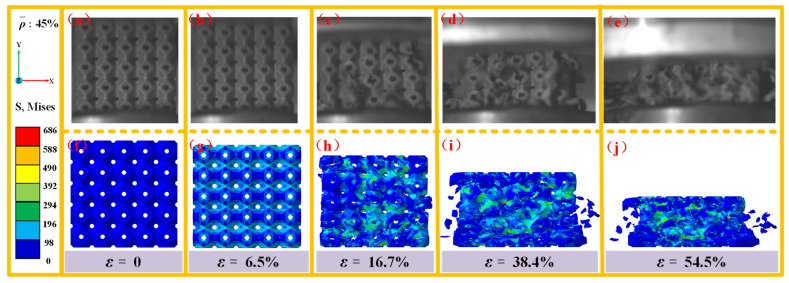
Comparison of experimental observations and simulations of the damage failure modes of SFRP porous structures with a volume fraction of 45% at medium strain rates. (**a**) experimental observation at ε = 0; (**b**) experimental observation at ε = 6.5%; (**c**) experimental observation at ε = 16.7%; (**d**) experimental observation at ε = 38.4%; (**e**) experimental observation at ε = 54.5%; (**f**) stress cloud for damage failure modes at ε = 0; (**g**) stress cloud for damage failure modes at ε = 6.5%; (**h**) stress cloud for damage failure modes at ε = 16.7%; (**i**) stress cloud for damage failure modes at ε = 38.4%; (**j**) stress cloud for damage failure modes at ε = 54.5%.

Figure 18 provides a comparison between the experimental observations and numerical simulations of the damage and failure modes in SFRP porous structures with a volume fraction of 45% under medium strain rate loading. At ε = 0, the structure remains in its initial unloaded state. When the strain reaches ε = 6.5%, the structure undergoes small, uniform elastic deformation along the loading direction (y-direction). As the strain increases to ε = 16.7%, irreversible elastic–plastic deformation begins, accompanied by the initiation of damage cracks around the pores. At ε = 38.4%, crack propagation intensifies, leading to rapid structural damage. The stress cloud maps from the numerical simulations reveal numerous stress concentration areas at this stage. By ε = 54.5%, the structure experiences complete fragmentation due to impact energy dissipation, resulting in splashing material fragments. High-stress zones are prominently observed in the numerical simulation maps, indicating the final stage of structural failure. Simulated stress contours for the 45% volume fraction structure (Figure 18) reveal stress distributions within the residual structure consistent with the experimental “blocky fragment” load-bearing behavior, demonstrating the model’s ability to capture the composite energy absorption mechanism (residual structure load-bearing–pore compression) at a high fiber content.

Notably, the critical strain for damage initiation in the 45% volume fraction SFRP porous structure is delayed compared to the structures with lower volume fractions (25% and 35%), demonstrating superior deformation resistance. Furthermore, the stress maps reveal a more intricate damage evolution path, suggesting that the failure process in the 45% volume fraction structure involves a more complex energy dissipation mechanism. Despite its enhanced deformation resistance, the strain interval from damage initiation to complete failure is reduced by approximately 20% compared to the lower porosity structures. This reduction is attributed to the accelerated damage accumulation rate caused by the higher relative density of fibers at increased porosity, which promotes faster stress interactions and damage development.

By comparing the experimental and numerical simulation results for SFRP porous structures with three different volume fractions (25%, 35%, and 45%) subjected to impact loading from a falling hammer at a medium strain rate, a high degree of consistency in the damage evolution process is observed. This strong agreement between the experimental and simulation outcomes validates the reliability and accuracy of the numerical model. Furthermore, as the volume fraction increases, the structures exhibit an enhanced initial load-bearing capacity and demonstrate more intricate damage patterns during failure. Higher volume fractions, characterized by denser fiber networks, contribute to greater resistance to deformation in the early loading stages, while promoting accelerated damage accumulation and more complex energy dissipation mechanisms during failure. This relationship underscores the significant influences of porosity and fiber density on the mechanical behavior and damage evolution of SFRP porous structures.

## 6. Conclusions

(1) At moderate strain rates, in SFRP porous structures with low fiber volume fractions, the peak stress increased with increasing strain rate, indicating the predominance of the strain rate strengthening effect. In contrast, for structures with high fiber volume fractions, competition between the strain rate strengthening effect and the damage evolution mechanism resulted in more complex stress–strain curve profiles. Specifically, the dominance of damage evolution led to the lowest peak stress at intermediate strain rates, whereas the superior performance of the strain rate strengthening mechanism at higher strain rates yielded the highest peak stress. Furthermore, across all three fiber volume fractions, damage accumulation was relatively slow and the stress rise was more gradual at lower strain rates, exhibiting distinct differences in curve morphology compared to those observed at moderate-to-high strain rates.
(2) For SFRP porous structures with low fiber volume fractions, both the SEA and EAE increased significantly with the rising strain rate, demonstrating a pronounced strain rate effect. In contrast, for structures with medium and high fiber volume fractions, the dual mechanisms of strain rate strengthening and damage evolution led to considerable variations in both SEA and EAE across different strain rates. This resulted in an alternating dominance of the energy absorption mechanisms and consequently more complex curve profiles.
(3) Under drop-weight impact, the deformation and failure modes of SFRP porous structures across all three fiber volume fractions were fundamentally similar. A higher impact energy induced rapid crack initiation and propagation, culminating in crushing dominated by shear failure. The microscopic damage morphology analysis at moderate strain rates revealed distinct characteristics dependent on the volume fraction. (i) For the 25 vol% structure, damage primarily manifested as fiber pull-out, matrix wrinkling, and tearing; (ii) the 35 vol% structure exhibited matrix plastic deformation, generating fibrillar structures accompanied by progressive interlaminar damage; and (iii) the 45 vol% structure demonstrated a coexistence of brittle fracture and plastic deformation.
(4) Numerical simulations demonstrated good agreement with the experimental observations regarding the damage and failure modes. The multidirectional cross-sectional failure analysis indicated that under impact loading, local shear instability occurred in the porous structures due to relative sliding along oblique planes. Furthermore, the primary reason for structural failure was attributed to the initiation of multiple shear bands and their subsequent rapid propagation.

## Figures and Tables

**Table 1:** The process parameters of Bambu Lab X1.

Process Parameters	Filament Diameter	Nozzle Diameter	Build Plate Temperature	Nozzle Temperature	Scan Speed	Layer Thickness	Annealing Temperature	Annealing Duration
Bambu Lab X1	1.75 mm	0.4 mm	100 °C	280 °C	500 mm/s	0.15 mm	80 °C	8 h

**Table 2:** Prediction of the equivalent elastic constants of the RVE unit-cell model.

Elastic Constant	*E*_11_/GPa	*E*_22_ = *E*_33_/GPa	*G*_12_ = *G*_13_/GPa	*G*_23_/GPa	*ν*_12_ = *ν*_13_	*ν*_23_
Finite element homogenization	3.040	3.124	1.126	1.134	0.349	0.340

**Table 3:** Mechanical performance parameters of RVE structures.

Materials	Elastic Modulus (GPa)	Shear Modulus (GPa)	Poisson’s Ratio
CF	230	30	0.3
PLA matrix	2.97	1.08	0.35

**Table 4:** Numerical simulation of the material parameters of the SFRP macro model.

Material Parameters	Values
Elastic Modulus *E_c_* (GPa)	3.11
Poisson’s Ratio *ν*	0.34
Tensile Strength *σ_t_* (MPa)	45.28
Compressive Strength *σ_c_* (MPa)	108
Shear Strength *τ* (MPa)	45

## Data Availability

The original contributions presented in this study are included in this article; further inquiries can be directed to the corresponding author.
